# Formins in Human Disease

**DOI:** 10.3390/cells10102554

**Published:** 2021-09-27

**Authors:** Leticia Labat-de-Hoz, Miguel A. Alonso

**Affiliations:** Centro de Biología Molecular Severo Ochoa, Consejo Superior de Investigaciones Científicas, Universidad Autónoma de Madrid, 28049 Madrid, Spain; llabat@cbm.csic.es

**Keywords:** formins, genetic disease, gene variants, developmental defects, cancer, actin

## Abstract

Almost 25 years have passed since a mutation of a formin gene, *DIAPH1*, was identified as being responsible for a human inherited disorder: a form of sensorineural hearing loss. Since then, our knowledge of the links between formins and disease has deepened considerably. Mutations of *DIAPH1* and six other formin genes (*DAAM2*, *DIAPH2*, *DIAPH3*, *FMN2*, *INF2* and *FHOD3*) have been identified as the genetic cause of a variety of inherited human disorders, including intellectual disability, renal disease, peripheral neuropathy, thrombocytopenia, primary ovarian insufficiency, hearing loss and cardiomyopathy. In addition, alterations in formin genes have been associated with a variety of pathological conditions, including developmental defects affecting the heart, nervous system and kidney, aging-related diseases, and cancer. This review summarizes the most recent discoveries about the involvement of formin alterations in monogenic disorders and other human pathological conditions, especially cancer, with which they have been associated. In vitro results and experiments in modified animal models are discussed. Finally, we outline the directions for future research in this field.

## 1. Introduction

**Figure 1:**
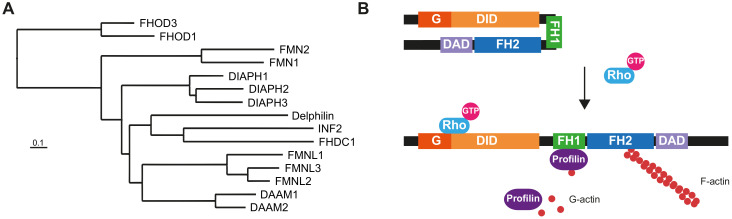
The human formin family. (**A**) Tree of human formins. The FH2 domain sequence of the formins was aligned with BLAST and the alignment was used to construct the tree [103]. The UniProt accession numbers of the corresponding sequences were: DIAPH1 (O60610), DIAPH2 (O60879), DIAPH3 (Q9NSV4), DAAM1 (Q9Y4D1), DAAM2 (Q86T65), FMNL1 (O95466), FMNL2 (Q96PY5), FMNL3 (Q8IVF7), FHOD1 (Q9Y613), FHOD3 (Q2V2M9), FMN1 (Q68DA7), FMN2 (Q9NZ56), INF2 (Q27J81), FHDC1 (Q9C0D6) and Delphilin (A4D2P6). (**B**) Structure and regulation of Diaphanous-related formins. The interaction of the DID and the DAD maintains the formin in a closed, inactive conformation. The binding of a specific GTP-loaded Rho GTPase to the N-terminal region of the formin opens the molecule, rendering it in its active form. The FH1 domain recruits profilin, which feeds the FH2 domain with G-actin to form the actin filaments. The illustrated molecules are not drawn to scale.

The human formin family consists of fifteen members (Figure 1A), divided into seven subfamilies [1], many of which are co-expressed in many tissues (Supplementary Tables S1 and S2). Formins are involved in the polymerization of monomeric actin into linear filaments [2,3]. All formins possess two characteristic domains: a formin homology (FH) 2 domain, which catalyzes actin polymerization, and an FH1 domain, which binds profilin to provide monomeric actin to the FH2 domain. The other regions and domains can differ between formin subfamilies and are involved in regulatory mechanisms or specific interactions with other proteins. In addition to regulating the actin cytoskeleton, formins bind to microtubules through the FH2 domain and regulate the acetylation and stability of microtubules, and their alignment with actin filaments [4,5].

Members of the human Diaphanous-related formin subfamily, which includes Diaphanous homolog (DIAPH) 1-3, are regulated through the interaction of the Diaphanous inhibitory domain (DID) at the N-terminal end and the Diaphanous autoregulatory domain (DAD) at the C-terminal region [1]. The transition between closed/inactive and open/active states is mediated by the interaction of the Rho-family GTPases with the DID, which releases its interaction with the DAD (Figure 1B). Other formins with similar regulation are Disheveled-associated activators of morphogenesis (DAAM) 1 and 2, formin-like (FMNL) 1-3, and FH1/FH2 domain-containing (FHOD) 1 and 3.

**Figure 2:**
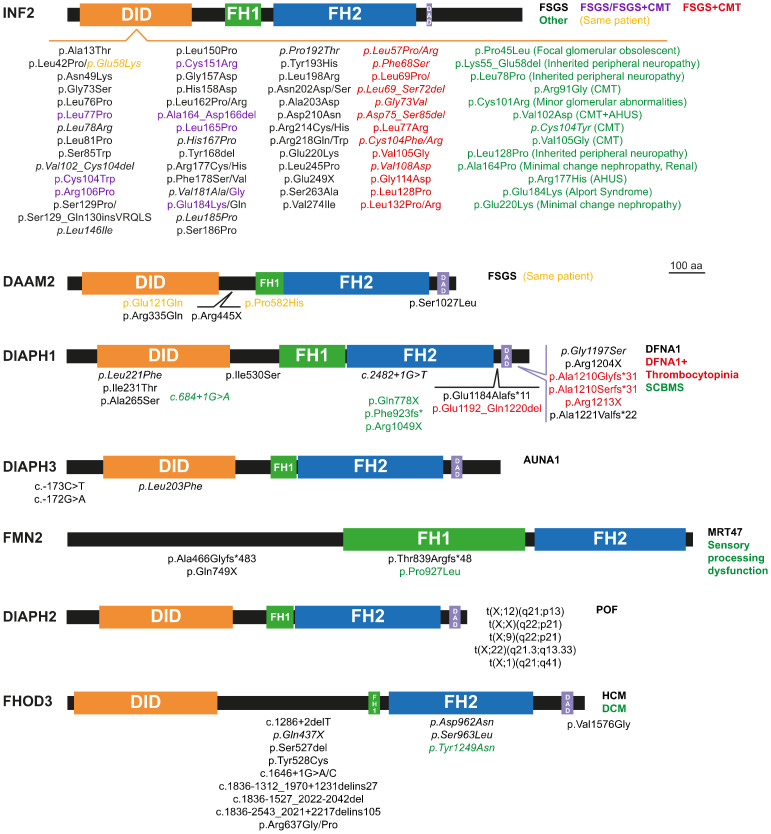
Pathogenic mutations of the formins causing monogenic disorders. Depending on the specific mutation, some formins produce different disorders. In these cases, we used the colors, as indicated, to refer to each of the diseases and the corresponding mutations. *, stop codon. The mutations without reported familial studies are indicated in italics.

**Figure 3:**
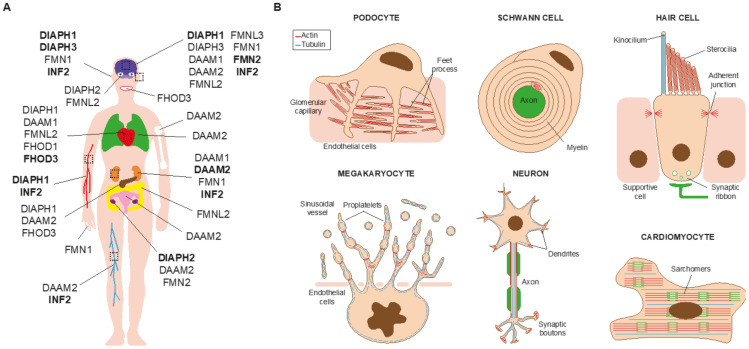
Some of the organs and cell types affected by formin alterations. (**A**) The formins involved in human disorders and the affected organs and systems are indicated in the schematic of the human body. Those causing monogenic disorders are highlighted in bold. (**B**) Some of the affected cell types. Their most characteristic structures are indicated.

The mutation of some of the formin genes causes monogenic disorders, as is the case of *DIAPH1*, which was the first formin gene found to be linked to a human Mendelian disorder [6]. Alteration of seven formins genes (Figure 2) have been acknowledged to date by the Online Mendelian Inheritance in Man (OMIM^®^, McKusick-Nathans Institute of Genetic Medicine, Johns Hopkins University (Baltimore, MD, USA), https://omim.org; accessed on 20 September 2021) as meeting the criteria for consideration as a primary cause of human monogenic disorders [7,8]: *DIAPH1-3* [6,9,10,11,12,13,14,15,16,17,18,19,20,21,22,23,24,25,26,27,28,29,30,31,32,33,34,35,36] (Supplementary Tables S3–S5), *DAMM2* [37] (Supplementary Table S6), *FORMIN2* [38,39,40,41,42] (*FMN2*) (Supplementary Table S7), *INVERTED FORMIN 2* (*INF2*) [43,44,45,46,47,48,49,50,51,52,53,54,55,56,57,58,59,60,61,62,63,64,65,66,67,68,69,70,71,72,73,74,75,76,77,78,79,80,81,82,83,84,85,86,87,88,89,90,91,92,93,94] (Supplementary Table S8) and FHOD3 [95,96,97,98,99,100] (Supplementary Table S9). The mutations or dysregulation of the other formins have not been demonstrated to be the primary cause of the phenotype, although they probably contribute to it [101,102]. Mutant formins can alter specific organs by affecting the functioning of specific types of cell (Figure 3A,B). Here, we review the involvement of formin genes in human monogenic disorders, comment on the association of other formin genes with similar or other disorders, and discuss the experimental evidence (from in vitro experiments, modified animal models, etc.) of their roles in disease.

## 2. Monogenic Disorders Caused by Formin Mutation

### 2.1. Nephrotic Syndrome and Charcot-Marie-Tooth Disease

Blood filtration and the concentration of metabolic waste into urine take place in the renal glomeruli. Podocytes are terminally differentiated cells that wrap around endothelial cells of glomerular capillaries by means of elaborate projections known as foot processes (Figure 3B). The contact between two of these processes forms a slit diaphragm, which is the structure responsible for blood filtration [104]. Deficient blood filtration causes nephrotic syndrome, which is characterized by proteinuria, hypoalbulinemia, hyperlipidemia, and edema, and can end in renal failure.

Focal segmental glomerulosclerosis (FSGS) refers to a histological lesion of scarred appearance present in localized regions of some, but not all, glomeruli [105]. The *INF2* gene is the formin for which the greatest number of pathogenic mutations has been described (Figure 2 and Supplementary Table S8) [106]. *INF2* pathogenic mutations are autosomal dominant and produce FSGS (FSGS5, MIM: 613237) [43], which causes steroid-resistant nephrotic syndrome [107,108]. Depending on the specific mutation, FSGS co-occurs (or not) with Charcot–Marie–Tooth disease (CMTDIE, MIM: 614455) [48], which is a neuropathy affecting the functioning of the peripheral nerves that produces progressive distal muscle weakness [109]. All the INF2 disease-related mutations localize to the DID and the great majority are of the missense type. Genomic-wide screening (GWS) and whole-exome sequencing (WES) analyses in patients with renal disease identified a number of variants outside the INF2 DID [106] but, with the exception of a case of FSGS combined with CMT with a deletion in the DAD [94], it is not clear whether these variants are related to the pathogenic condition.

An in silico analysis of the effect of the pathogenic mutations indicates that they have a destabilizing structural effect in the DID [106]. This destabilization might affect the interaction of the DID with the DAD or with regulatory proteins [110,111], and results in gain-of-function of the actin polymerization activity of INF2. The case of a patient with combined FSGS and CMT has been described, in which a complete duplication of the *INF2* gene occurred, which represents further evidence of a gain-of-function phenotype in INF2-linked disease [93]. It is of note that the mutations causing combined FSGS and CMT are generally more destabilizing than those producing only FSGS, and that these two types of mutation distribute in the DID in a different manner, with the former being concentrated in the N-terminal half of the DID, whereas those causing only FSGS are distributed throughout the DID [106].

FSGS patients suffer a progressive loss of podocytes, which decreases the filtration capacity of the kidney. INF2-linked FSGS starts to become clinically relevant in adolescence or adulthood, causing glomerular dysfunction [107,108]. It is still not clear how INF2 mutations affect podocytes but, consistent with the enhanced actin polymerization activity of the pathogenic INF2 mutants [110], aberrant actin bundles have been observed in a renal biopsy of an affected patient [43]. Knock-in mice expressing the most common mutation, p.Arg218Gln, exhibit no apparent alteration in podocyte structure unless they are exposed to acute kidney injury [112]. This finding is consistent with the degenerative nature of INF2-related disease and suggests that FSGS might be the result of repeated kidney insults in individuals in which INF2 mutation makes them more prone to developing the disease. In addition to FSGS, *INF2* mutations have been found to contribute to, or be responsible for, other kidney conditions (Figure 2 and Supplementary Table S8). In patients with combined FSGS and CMT, CMT symptoms appear in childhood, and renal damage appears earlier in life than in patients with only FSGS. In the cases with CMT, pathogenic INF2 affects Schwann cell polarization (Figure 3B), leading to abnormal myelin formation and/or maintenance [113,114]. The manifestation of FSGS alone is common in individuals with pathogenic INF2, but only one case of CMT has been described to date that makes the absence of accompanying renal disease explicit [59].

Recessive mutations of *DAAM2* (Figure 2 and Supplementary Table S6), have recently been involved in nephrotic syndrome, type 24 (NPHS24, MIM: 606627) [37]. All the affected individuals presented FSGS with no extra-renal manifestations. The mutations were found in homozygosity in three individuals from consanguineous families, and in one individual with two different missense mutations from an outbred family. The missense mutations map to the region encoding the DID, the FH1 or the DAD, whereas the nonsense mutation maps immediately downstream of the DID and generates a truncated DAAM2 protein. The mutations at the DID and DAD appear to cause increased autoinhibition and, consequently, loss-of-function of actin polymerization activity. DAAM2, which is expressed by podocytes, colocalizes and associates with INF2 [37], suggesting the existence of crosstalk between the two formins that, given the link between INF2 and renal disease, may explain the renal damage caused by pathogenic DAAM2. Other formins might be involved in other kidney disorders. For instance, Fmn1 has recently been identified as a candidate modifier gene in X-linked Alport syndrome in mice, which is a genetic disease characterized by hearing loss, hematuria and, eventually, renal failure [115].

### 2.2. Hearing Loss

Hearing depends on the correct mechanostransduction of sound vibrations into electrical signals. This takes place in the organ of Corti, which is located in the cochlea in the inner ear. The cells responsible for this process are sensory epithelial cells, known as hair cells (Figure 3B), which possess dozens of stereocilia on their apical surface, formed of bundles of actin filaments [116]. Outer hair cells amplify the signal, and the perturbation activates the opening of ion channels at the stereocilia tips of inner hair cells, depolarizing the plasma membrane. This perturbation is subsequently transmitted by neurotransmitters released at the synaptic ribbon between the basolateral surface of hair cells and the auditory nerve. This generates electrical impulses in the latter that are transmitted to the brain, where they are decoded and analyzed in the auditory cortex. Given the importance of actin in the architecture of stereocilia, mutations in actin, actin-binding proteins, and the machinery involved in actin filament formation and function, including formins, can all cause hearing loss [117].

Sensorineural hearing loss is caused by dysfunction of the inner ear or the auditory nerve. Mutations in *DIAPH1* produce deafness, autosomal dominant 1 (DFNA1, MIM: 124900). In this disorder, auditory loss generally starts during the first decade of life, although there are cases with intrafamilial variability. Since the identification of a mutation in *DIAPH1* as the cause of sensorineural hearing loss in a large Costa Rican family [6], more families with a dominant pedigree caused by *DIAPH1* mutation have been described elsewhere in the world [11,12,13,18,19,21,22,23,24,25,26,27]. Affected individuals present frameshift or nonsense mutations or deletions near the DAD. These types of mutation create truncated forms of DIAPH1 that lack different segments of the carboxyl-terminal region of the molecule. In addition, more recently, missense mutations have been described at the DID and the coiled-coil downstream region and FH2 domain (Figure 2 and Supplementary Table S3). The pathogenic p.Arg1204X mutation [21] results in early termination immediately before a basic amino acid motif (RRKR^1204–1207^) present at the DAD C-terminus, which is important for the interaction with the DID [118]. This mutation partially relieves the autoinhibitory DID-DAD interaction, resulting in a mildly constitutive active molecule [21]. It is likely that this also occurs with other truncation mutations mapping around this site and with the missense mutations in the DID [119,120]. The *DIAPH1* gene mouse homolog, *mDia1*, is expressed in the organ of Corti during and after cochlear maturation, and localizes at the apical junctional complexes between the supporting cells and the hair cells [121]. As further evidence that hearing loss caused by *DIAPH1* mutations is due to gain- and not to loss-of-function, hearing progressively deteriorates in transgenic mice overexpressing wild-type mDia1 [121], whereas the hearing function of the *mDia*1 knock-out (KO) mice is not different from that of control mice [21]. The hearing defect in mice overexpressing mDia1 is associated with gradual loss of hair cells and the appearance of sparse and short or fused stereocilia cells [121]. A similar phenotype was observed in transgenic mice expressing the human Arg1204X mutant [21]. Increased gene dosage of *DIAPH1* has been documented in several cases of sporadic sensorineural hearing loss in humans [28]. These findings are further evidence that deafness-associated mutations of DIAPH1 cause disease by increasing actin polymerization activity, which causes the disorganization and dysfunction of stereocilia.

Auditory neuropathy, autosomal dominant, 1 (AUNA1, MIM: 609129) is characterized by abnormal or absent auditory brainstem responses but preserved cochlear outer hair cell function. A mutation (c.-172G > A) in a highly conserved GC element at the exon encoding the 5′ untranslated region of *DIAPH3*, was the first to be described as being involved in AUNA1. This mutation, which is probably of the gain-of-function type, results in 2- to 3-fold overexpression of *DIAPH3* mRNA and 1.5-fold overexpression of DIAPH3 protein levels. Consistent with increased levels of DIAPH3 as the cause of the auditory alterations, flies expressing a constitutively active form of Diaphanous, which encodes the sole Diaphanous-related formin in *Drosophila,* show an impaired response to sound [36]. Two reports found that mice overexpressing mouse mDia2, the murine homolog of human DIAPH3, present progressive impairment of inner hair cell stereocilia, whereas outer hair cells stereocilia and function were not generally affected in the specific mouse lines studied [122,123]. A reduction in the number of ribbon synapses was observed in one study [122], but not in the other [123]. Consistent with the role of formins in regulating microtubule dynamics, the microtubule meshwork undergoes aberrant targeting to the apical aspect of inner hair cells in transgenic mDia2 mice [123], probably contributing to stereocilia collapse. These mice also present early mortality due to cardiac defects, but no similar effect has yet been found in humans. In addition to the c.-172G > A mutation, other mutations causing AUNA1 have been described at the 5′ untranslated region of *DIAPH3* mRNA [35] and the DID of DIAPH3 [15] (Figure 2, Supplementary Table S5). Missense variants mapping to the DIAPH3 FH2 domain have also been found in patients with auditory neuropathy spectrum disorders [15,124], but it is not clear whether there they are pathogenic or simply rare variants.

In the case of *INF2* mutations associated with combined CMT plus FSGS, but not with FSGS alone, some of the patients also experience hearing loss [48,49,51,57]. Since INF2 mutations causing CMT affect peripheral nerve myelinization [113], auditory nerve damage is probably the cause of the hearing impairment, although hair cell stereocilia may also be affected, as in cases of *DIAPH1* and *DIAPH3* mutations.

### 2.3. Thrombocytopenia

The cell precursors of platelets, megakaryocytes, form extensions known as proplatelets, from which platelets are released into the circulatory system [125]. Macrothrombocytopenia is characterized by enlarged and reduced numbers of circulating platelets that can lead to inadequate clot formation and an increased risk of bleeding [125]. Platelet production begins with the extension of long membrane protrusions that are elongated by microtubule bundles to form proplatelet processes (Figure 3B). Amplification of the number of processes, which occurs by repeated bending and bifurcation, depends on actin filament formation [126,127,128]. It is controversial whether actin/microtubule crosstalk-induced proplatelet formation [126] or membrane budding without requiring proplatelet formation is the main mechanism of platelet formation in vivo [129].

Long after the discovery of the *DIAPH1* mutation as the cause of DFNA1, affected individuals were found to present asymptomatic thrombocytopenia and, sometimes, asymptomatic mild neutropenia (Figure 2, Supplementary Table S3), which consists of abnormally low levels of neutrophils in the blood. The reduced platelet levels in these patients, the high content of polymerized actin, and the altered microtubule organization and stability observed in the platelets [27] are consistent with the requirement of DIAPH1 for proper proplatelet formation [130,131], and with previous works showing that DIAPH1 coordinates microtubules and the actin cytoskeleton [132,133]. It is of note that a moderate increase in the expression of DIAPH1 could be responsible for the thrombocytopenia associated with diseases caused by mutation in other genes, as may be the case for Roifman syndrome [134]. This is a rare, inherited disease (MIM: 616651) characterized by growth retardation, cognitive delays, skeletal malformations and immunodeficiency, and caused by mutation of the small non-coding RNA gene *RNU4TAC* [135].

Atypical hemolytic uremic syndrome (AHUS) is characterized by acute renal failure, thrombocytopenia, and microangiopathic hemolytic anemia (loss of blood cells through destruction). Two mutations in the DID of INF2 cause thrombocytopenia in the context of familial AHUS with (p.Val102Asp) or without (p.Arg177His) associated CMT [60]. In AHUS, the thrombocytopenia is due to platelet activation and consumption associated with blood cell destruction, rather than to an alteration in platelet production.

### 2.4. Microcephaly and Intellectual Disability

According to the Human Protein Atlas (http://www.proteinatlas.org; accessed on 30 August 2021), and consistent with the analyses of mouse brain [136], all the formins are expressed throughout the brain, generally with low regional specificity (Supplementary Table S2). Mutations of *DIAPH1*, *FMN2,* and *INF2* are associated, to varying degrees, with intellectual disability and neurodevelopmental disorders [137].

*DIAPH1* is expressed in neuronal progenitors during brain development [14]. Specific mutations of *DIAPH1* cause seizures, cortical blindness (vision loss due to a damage or malfunction in the part of the brain cortex responsible for processing visual information), and microcephaly syndrome (SCBMS, MIM: 616632) (Figure 2, Supplementary Table S3). In contrast to DFNA1-related mutations, SCBMS-associated *DIAPH1* mutations are generally of the nonsense type that affects the FH2 domain, are found in homozygosity, and are inherited with an autosomal recessive pattern, suggesting that they produce loss-of-function of DIAPH1 activity [10,14,16,17]. *mDia1* KO mice are not microcephalic but, instead, some mice present unilateral dilatation of the ventricles, indicating that the effect of these mutations is species-specific [14]. Unlike *mDia1* KO mice, *mDia2* KO mice present microcephaly and also hydrocephalus (accumulation of cerebrospinal fluid within the brain) [138]. This phenotype seems to be due to incorrect spindle assembly checkpoint regulation in cortical progenitor cells, causing massive loss of cortical progenitor cells, with the subsequent depletion of neurons [138]. *mDia1* and *mDia3* double-KO mice present hydrocephalus, but not microcephaly, due to the formation of a periventricular dysplastic (abnormal) mass during brain development [139]. The alteration of the actin cytoskeleton affecting the adherens junctions and progenitors’ polarity seems to be the cause of the ectopic proliferation of neural stem cells in the double-KO mice. In addition to the characteristic SCBMS symptoms, some patients present pathologies related to immunodeficiency, such as recurrent infections, especially respiratory, bronchiectasis (enlargement of parts of the airways of the lung) and lymphoma [10,14,16]. Given that (i) *mDia1* KO mice show defects in T cell migration and activation [140,141], (ii) *DIAPH1* mutations are associated with mitochondrial dysfunction [10], and (iii) fibroblasts and some lymphocytes from SCBMS patients present mitochondrial alterations [10], it has been proposed that these additional symptoms are due to a defect in the mechanism of T cell activation [10].

A few cases of intellectual disability denominated mental retardation, autosomal recessive 47 (MRT47, MIM: 616193), are produced by mutations of *FMN2* [38,39,41,42] (Figure 2, Supplementary Table S7). The genomic alterations consist of homozygous frameshift and nonsense mutations that are always found in consanguineous families, and large de novo heterozygous deletions. One case with sensory processing dysfunction was also associated with a de novo missense mutation [40]. This phenotype is consistent with the role of FMN2 in stabilizing filopodia tip adhesions and regulating the chemotaxis of neuronal grown cones [142,143]. Unlike the effect of *FMN2* mutations in humans, *Fmn2* KO mice do not present any alteration in the brain [144]. However, double-KO mice of *FMN2* and *filamin A* show greater microcephaly severity and less neural progenitor proliferation compared with the phenotype of single *filamin A* KO mice. It has been suggested that this additive effect is a consequence of FMN2 and filamin A both forming part of the machinery of the endocytic route of the canonical Wnt pathway that regulates neural progenitor proliferation [145].

Mutations of other formin genes in addition to *DIAPH1* and *FMN2* have been associated with intellectual disability. In the case of *INF2*, some patients with FSGS and associated CMT, probably with severe mutations or an unfavorable genetic background, present intellectual disability and central nervous system anomalies [48,49,51]. An almost complete deletion of the *FMNL2* gene has been associated with a case of mental retardation [146]. However, since the patient also presented haploinsufficiency in *NR4A2*, a gene involved in the cerebral dopaminergic system, it is difficult to ascertain whether the disorder is caused by one or both mutations.

### 2.5. Primary Ovarian Insufficiency

*DIAPH2* has been implicated in premature ovarian failure (POF2A, MIM: 300511), also known as primary ovarian insufficiency, which manifests as premature menopause [29,30,31,32,33,34]. The patients generally present translocations of the X chromosome region that includes *DIAPH2* (Figure 2, Supplementary Table S4). This gene might be involved in the development of gonads since it is expressed in the ovaries and testes of mouse embryos [29], and, indeed, some of the patients present ovarian dysgenesis (abnormal development) [33,34]. Underlining the importance of DIAPH2, *Drosophila* with mutations in *Diaphanous* are sterile due to cytokinesis failure that affects spermatogenesis in males and follicle cell division in females [147].

Consistent with the possibility that formin genes other than *DIAPH2* are related to POF, *FMN2* has been associated with POF and infertility in mice and in human patients [148,149]. Female *Fmn2* KO mice exhibit defects in spindle positioning during meiosis I [144], which explains their low fertility. It has been proposed that upregulated levels of FMNL2 in humans also have a role in female infertility and gynecological health since they promote adenomyosis, which is characterized by the ectopic growth of the endometrium in the uterine walls, which are formed by the myometrium [150].

### 2.6. Cardiomyopathy

Thirteen out of the fifteen formins are expressed during postnatal development of the heart in mice within a specific timeframe that suggests a role for each formin in this process [151]. *FHOD3,* which is mainly expressed in the heart and regulates actin assembly in cardiomyocytes [152] (Figure 3B), has been linked to cardiac pathologies [153]. *FHOD3* mutations have been associated with hypertrophic (CMH28, MIM: 619402) [95,96,97,98,99,100] and dilated cardiomyopathies [154] (Figure 2, Supplementary Table S9), which are conditions in which the walls of the heart becomes thicker and stiff, and where the heart is enlarged, respectively. As a consequence of these alterations, blood is pumped less effectively. Two intronic variants of *FHOD3* have also been related to hypertrophic cardiomyopathy development [155] and a conservative substitution (p.Val1151Ile) with a reduced risk of dilated cardiomyopathy [156]. *Fhod3* KO mice present embryonic lethality due to defects in cardiogenesis and in neural tube closure [157], whereas conditional KO mice show that the FHOD3 protein is needed not only for prenatal and postnatal heart development, but also for its maintenance, since adult mice present cardiomegaly and mild impairment of cardiac function [158]. Transgenic mice expressing FHOD3 defective in actin binding have a similar phenotype to that of dilated cardiomyopathy patients [157]. It is likely that the specific domain affected by the mutation, as well as the individual genetic background, could determine the appearance of one or other pathology, although both appear to be inherited in an autosomal-dominant manner. Angiotensin II is an important factor causing blood pressure overload-induced cardiac hypertrophy [159]. In cultured rat cardiomyocytes, angiotensin II signaling regulates FHOD3 activation through phosphorylation of its C-terminal region by ROCK kinase, raising the possibility that pathogenic FHOD3 causes heart hypertrophy by this mechanism [160].

## 3. Disorders Associated with Formin Mutation and Dysregulation

### 3.1. Developmental Cardiovascular Disorders

Myotonic dystrophies are multisystemic inherited disorders characterized by progressive muscle weakness, myotonia (prolonged muscle contraction), cardiac defects and early cataracts. Myotonic dystrophic types 1 (DM1; MIM: 160900) and 2 (DM2; MIM: 602668) are caused by the expansion of a trinucleotide (CTG)n or a tetranucleotide (CCTG)n in the 3′ untranslated region of, respectively, the *dystrophia myotonica protein kinase* (*DMPK*) and the *nucleic acid binding protein* (*CNBP*) genes, respectively [161]. The expression of the expanded transcripts causes aberrant splicing of many genes involved in muscle homeostasis and function [162]. FHOD1, the other member of the FHOD family, has not yet been associated directly with any human disease, but *FHOD1* transcripts are known to undergo aberrant splicing in DM1 [163] and DM2 [164], raising the possibility that the alteration of *FHOD1* is somehow involved in impaired cardiac function in these two diseases.

Deletions affecting *DAAM1* have been implicated in congenital heart defects in humans [165,166]. *Daam1* is expressed in the heart during murine embryonic development [167]. *Daam1* KO mouse embryos present abnormalities that affect heart morphogenesis, and the cardiomyocytes exhibit aberrant organization of the actin cytoskeleton, fewer, underdeveloped sarcomeres (structures containing crosslinked bundles of actin filaments and interdigitating myosin filaments), and cell–cell junction defects [167].

### 3.2. Neurodevelopmental Disorders and Mental Diseases

Formin mutation has been linked not only to intellectual disability, but also to several neurodevelopmental disorders [137]. Autism spectrum disorder (ASD, MIM: 209850) is a neurodevelopmental condition characterized by young age of onset, impairment of communication and social abilities, and restricted interests and repetitive behaviors. The interaction between genetic and environmental conditions plays an important role in establishing this disorder [168]. *DIAPH3* seems to be the formin gene most strongly linked to autism, as suggested by the identification of variants in autistic individuals. The first case to relate *DIAPH3* with autism was that of a boy who inherited a variant (p.Pro614Thr) in the FH1 domain of one of the alleles and a large genomic deletion in the other, *DIAPH3* being one of the genes considered in the study [169]. This double-hit case is very rare and indicates that, in the absence of a double mutation in *DIAPH3*, at least one extra mutation may be needed in one or more autism-related genes to produce the phenotype. This was found in a second case that presented a missense replacement in the region of *DIAPH3* encoding the FH2 domain and a second mutation in *SET2* [170], a gene associated with autism and other neurodevelopmental disorders. Another example was a case with infantile spasm that presented a missense mutation in *DIAPH3* (p.Arg432Lys) and a nonsense mutation in the *syntaxin-binding protein 1* gene (*STXBP1*) [171], whose mutation is known to cause developmental and epileptic encephalopathy 4 (MIM:602926) [172]. Another interesting case was that of a patient with a rare epileptic encephalopathy featuring a deletion at 13q21 that included part of *DIAPH3* [173]. The specific KO of *Diaph3* in the brain cortex of mice alters the spindle components, leading to fewer proliferative divisions and more neurogenic divisions in apical neural progenitor cells, and thereby to a thinner cortex (microcephaly). Externally, these mice show motor activity deficiency and autism-related behavior [174].

An *FMN1* variant producing a missense substitution (p.Arg109Gly) was found in a search for genetic variants in high-risk ASD families [175]. A microdeletion affecting two exons of *FMN1* was found in a whole-genome amplification study of copy number variation in patients with early-onset obsessive-compulsive disorder [176]. An *FMN1* variant producing a missense mutation (p.Ser800Cys) was identified by WGS in a selected group of patients with schizophrenia. It displayed long homozygous runs throughout the genome due to recent inbreeding [177]. The possible involvement of *FMN1* mutations in these mental disorders could be explained by the role of FMN1 in the glutamatergic synaptic terminals [178].

In a study of Wnt-pathway-related genes in genome regions previously linked to schizophrenia, six variants were found in *DAAM2* [179]. *DAAM2* mRNA levels were significantly higher in blood samples from patients suffering their first psychotic episode than in controls, and returned almost to normal when the patients entered remission [180]. Guillain–Barre Syndrome (GBS, MIM: 139393), acute inflammatory demyelinating polyneuropathy, is most commonly characterized by symmetric limb weakness and loss of tendon reflexes. DAAM2 protein levels are increased in the acute phase of GBS, and their reduction is associated with the improvement of symptoms [181]. Given the role of DAAM2 in Wnt signaling, it was proposed that its levels alter the nuclear transport of critical transcriptional factors involved in myelination. However, the role, if any, of *DAAM2* in this group of disorders is currently unclear.

Two variants of *FMNL3* that produce missense substitutions (p.Phe38Leu and p.Ile458Leu) were identified in a consanguineous family containing siblings with an infancy-onset, mixed central and peripheral neurodegenerative disorder. However, since they present a homozygous mutation in the *STXBP5L* gene, this alteration was considered the most likely cause of the phenotype [182], although a contribution from the *FMNL3* variants cannot be ruled out.

### 3.3. Other Developmental Defects

Planar cell polarity (PCP) refers to the coordinated organization of cells or cell components across a tissue plane [183]. Defective PCP signaling is involved in the pathogenesis of developmental kidney disorders [184]. PCP is considered a noncanonical Wnt pathway (PCP/Wnt) due to the involvement of the secreted Wnt glycoproteins and the Frizzled family of Wnt receptors in the absence of the β-catenin-driven gene expression observed in the canonical Wnt cascade [185]. In the PCP/Wnt pathway, DAAM1 forms a complex with Disheveled that activates Rho, leading to cytoskeletal rearrangement [186]. In addition to the congenital heart defects mentioned above, *DAAM1* gene variants encoding a missense replacement (p.Val233Met) and a deletion (p.Lys681_Val682del) were identified in an analysis of a set of genes selected by their possible involvement in developmental anomalies of the kidney and urinary tract [187]. A duplication of exons 1-4 of *DAAM1* was identified in a study aimed at identifying copy number variants in cerebral palsy patients [188]. The developmental defects caused by the alterations in *DAAM1* could be explained by the defective functioning of the PCP/Wnt pathway [189]. Consistent with the importance of *DAAM1* in kidney development, DAAM1 knockdown (KD) impairs correct pronephric tubulogenesis in zebrafish and *Xenopus laevis* [190]. DAAM1 KD produces loss of primary cilia in kidney-derived cell lines [191], raising the possibility that the effect of reduced levels of DAAM1 on tubulogenesis is due to defective ciliogenesis. However, the effect on ciliogenesis is not observed in *Xenopus* nephric progenitor DAAM1 KD cells [190], making this explanation unlikely. Since *DAAM1* is also expressed in the neural tubule during embryonic development [192], *DAAM1* alterations could also affect developmental processes involving the PCP/Wnt pathway other than kidney tubulogenesis.

Similar to *DAAM1*, *DAAM2* has been linked to pathologies that might involve dysregulation of the PCP/Wnt pathway. A variant (p.Arg987Leu) in the FH2 domain of DAAM2 has been found in a sporadic case of diffuse pulmonary ossification, which is a disorder characterized by unusual widespread bony metaplastic formations in the lung [193]. Since osteoclasts strongly express DAAM2, but not DAAM1, the mutation could alter bone resorption via Wnt/DAAM2 [194]. A duplication of *DAAM2* was found in a family with a disorder of sexual development [195]. Since gene duplications and a mutation affecting other genes were also present, it is not possible to ascertain the involvement of *DAAM2* in this disorder. One possibility is that *DAAM2* is involved in gonadal development.

Limb development is a highly integrated process in which the cells involved must incorporate a variety of signals in a precise temporal manner. Defects in this process cause malformations in the extremities, as occurs Cenani–Lenz syndrome (MIM: 212780), which is a rare autosomal recessive disorder characterized by a complex syndactyly (two or more digits fused together) of the hands with malformations of the forearm bone and less severely affected lower limbs [196]. A de novo tandem duplication of the whole FMN1 gene was found in a patient with Cenani–Lenz-like non-syndromic oligosyndactyly. The same report described a homozygous deletion that affects the first twelve exons and the upstream region of *FMN1* in a consanguineous family with similar malformations and with hearing loss [197]. However, it seems that those defects are not caused by the change in the *FMN1* gene itself, since the altered genomic region also contains limb-specific regulatory sequences of *GREM1* [198], which is a gene involved in kidney and limb development. Although *Fmn1* KO mice in which the *GREM1* gene was not affected had a phenotype very similar to that of the patient with a homozygous deletion of *FMN1* [199], those mice had amplified *GREM1* expression, suggesting that *FMN1* regulates *GREM1*, and that the loss of *FMN1* is not the direct cause of the disorder. It has also been reported that *FMN1* in three patients with congenital anomalies of the kidney and urinary tract feature heterozygous missense (p.Arg752Trp or p.Pro529Arg) or frameshift (p.Ser1360Mfs*X19) variants [187]. However, the involvement of *GREM1* expression in this phenotype cannot be discounted.

### 3.4. Aging-Related Diseases

Age-related macular degeneration, which is a major cause of blindness, involves the progressive degeneration of photoreceptors and retinal pigment epithelial cells. In two studies of X-chromosome-linked variants, several variants associated with age-related macular degeneration were found in *DIAPH2* [200,201].

It was proposed that patients suffering neuropsychiatric disease, such as post-traumatic stress disorder (PTSD), at a young age have an increased risk of developing Alzheimer disease later in life [202,203]. Blood samples from PTSD patients and post mortem hippocampus samples from Alzheimer patients feature lower levels of *FMN2 mRNA* compared with controls [204], suggesting a possible association between these conditions and FMN2 downregulation. Consistent with this idea, it was observed that old *Fmn2* KO mice were more likely to show early age-associative memory impairment compared with age-matched controls, suggesting that reduced FMN2 levels contribute to age-related memory impairment [205]. However, the significance of this association is not clear because FMN2 is expressed normally in old mice [205]. Higher levels of the DIAPH1 protein were found in myeloid cells from the brain of Alzheimer patients compared with age-matched controls, and it was suggested that this increase could potentiate neuroinflammation via advanced glycation end (AGE) receptor (RAGE)/DIAPH1 signaling, thereby contributing to the disease [206,207].

### 3.5. Other Disorders

POEMS syndrome is a rare, multisystem disorder that may include polyneuropathy, organomegaly, endocrinopathy, and other features, including aberrant plasma cells. A frameshift insertion and a nonframeshift deletion of *FMNL2* were found by the WES of plasma cell DNA from a patient, although the role of these changes in the disease was not investigated [208]. Crohn’s disease is characterized by inflammation of parts of the digestive system and is associated with a failure of the innate immune system. Although the etiology of Crohn’s disease has not been fully determined, it clearly depends on genetic and environmental factors [209]. A heterozygous de novo mutation in the DID of FMNL2 (p.Leu136Pro) was found in a pediatric patient [210]. The mutation reduces the interaction between the DID and the DAD, with the mutant being more active than the wild-type protein. The stable expression of the mutant in THP-1 monocyte cells produces an increase in the number of podosome-like structures and a reduction in the percentage of THP-1 cells degrading the gelatin matrix in in vitro assays [210]. It is therefore plausible that FMNL2 regulates important processes dependent on actin dynamics in the cells of the innate immune system, since it is part of the genetic network that regulates the appearance of Crohn’s disease.

High levels of blood glucose stimulate the formation of AGE products, which are the result of non-enzymatic glycation and oxidation of proteins and lipids. AGEs accumulate in diabetic circulation and tissues and their interaction with RAGE, contributes to vascular alterations. The cytoplasmic domain of RAGE binds to DIAPH1, which is essential for RAGE ligand-mediated signal transduction [211]. It has been suggested that DIAPH1 could have a role in diabetes and obesity through its interaction with RAGE [212]. Indeed, studies in *mDia1* KO diabetic mice suggest a role of DIAPH1 in the pathogenesis of diabetes-associated nephropathy [213]. *FHOD3* mutations have been associated with type I diabetes [214], but further studies are needed to establish the relationship more convincingly. DAAM2 levels proved to be higher in diabetic nephropathy patients and in a mouse model of the disease [215], implying that a variety of formins may be involved in the diabetic condition.

*DAAM2* is expressed throughout pregnancy, reaching its highest levels at term. Pregnancies with a fetal growth restriction complication have raised blood levels of *DAAM2* mRNA and DAAM2 protein in preterm placental tissue [216]. This upregulation has been related to hypoxia, in which DAAM2 downregulates the expression of cytoprotective genes. The downregulation of *DAAM2* may thereby have a beneficial effect during this type of abnormal pregnancy. *DAAM1*, but not *DAAM2*, KO mice are lethal during embryogenesis due to placental development defects [217]. *Daam1* and *Daam2* double-KO mice have a more severe phenotype, suggesting redundant functions of *Daam* formins in this process [217].

Three intronic variants found within a 27-base pair region and two missense variants of *DAAM2* have been associated with osteoporosis [218,219]. As evidence for the role of DAAM2 in this process, it was found that hypomorphic Daam2 mice develop increased cortical porosity and reduced bone strength [218].

Ischemic stroke or brain ischemia is caused by a blockage in an artery supplying blood to the brain that reduces the flow of blood. Depending on whether circulation is restored, brain damage can be transient or permanent. Several formin genes are important in ischemic stroke. Thus, *FMNL1* and *FMNL2* appear to have a role in repairing damaged cardiomyofibrils [151]. This role might be evolutionary conserved since *FMNLs* genes are also expressed during heart development in zebrafish [220]. Consistent with their importance in this process, two *FMNL2* variants were identified in a genome-wide association analysis aimed at identifying genetic loci predisposing to early-onset stroke. An intronic variant in *DIAPH1* was associated with increased risk of ischemic stroke [221] and some *DIAPH1* variants have also been associated with Moyamoya disease [222], which is a progressive vasculopathy that causes stroke in children. The possible role of altered *DIAPH1* in ischemic stroke could be related to the involvement of RAGE/DIAPH1 in neuroinflammation and neurodegenerative diseases. Revascularization therapy is the primary modality for restoring the blood supply to infarcted brain tissues, but ischemia-reperfusion injury does run the risk of damaging the brain, worsening the stroke outcome. In cardiac ischemia-reperfusion injury, INF2 increases mitochondrial fission and represses mitochondrial fusion [223,224], contributing to cardiomyocyte death.

With regard to other variants of formin genes, several variants of *FHOD3* have been associated with periodontal disease [225]. In addition, several *FMNL2* variants have been associated with intraocular pressure and the subsequent development of glaucoma [226]. The latest finding could help explain some of the differences in glaucoma development observed between different ethnic populations [227].

## 4. Formins in Cancer

### 4.1. Expression of Formins in Cancer

According to the Human Protein Atlas (Supplementary Table S10), the mRNA levels of the different formins are generally of low specificity with regard to the type of cancer. However, *DAAM2* and *FMN1* expression is enhanced in melanoma, and that of *FMN2* in glioma and testicular cancer. Based on ONCOMINE^TM^ data (https://www.oncomine.org; accessed on 30 August 2021; Supplementary Table S11), *DIAPH1*, *DIAPH3*, *FMNL2, FHOD1,* and *INF2* transcripts are frequently upregulated in cancer, whereas those of *DIAPH2*, *DAAM1*, *DAAM2*, *FHOD3*, *FMN2,* and *FHDC1* are generally downregulated. The other formins are upregulated or downregulated, depending on the type of cancer. Most reports are of cancers in which the expression of a given formin gene or protein is upregulated, although there are some exceptions, such as the DIAPH3 and FMNL2 proteins, in which the expression is downregulated in triple-negative breast cancer and hepatocellular carcinoma, respectively (Table 1).

In some cases, the expression levels of formin mRNA have been linked to the methylation status of their promoters. For instance, *FHOD3* mRNA overexpression in thyroid cancer is associated with hypomethylation of the *FHOD3* promoter and with gene copy number variation [315]. Another example is the downregulation of *FMN2* expression by hypermethylation of its promoter during the early stages of colorectal cancer progression [269], possibly contributing to the initial steps of cancer development. It is also plausible that some formins can regulate the expression/activity of other formins. For instance, FMNL1 KD in glioblastoma cell lines downregulates DIAPH1 protein levels, whereas FMNL1 overexpression upregulates them [236], enabling malignant cells to migrate slower of faster, respectively, than control cells. Formins might also affect cancer progression through the expression of isoforms. For instance, in melanoma cell lines, the *FMNL2* gene encodes different isoforms with distinct abilities to promote invasion [308].

### 4.2. The Role of Formins in Cancer

**Figure 4:**
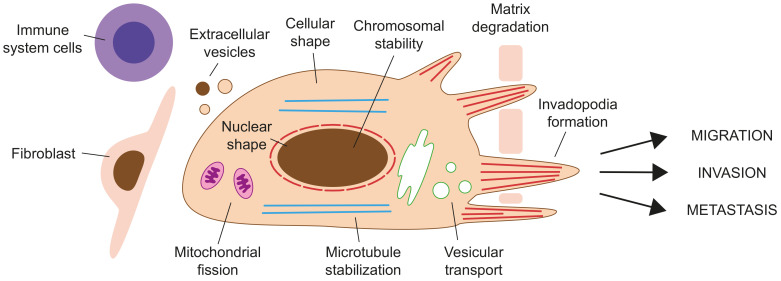
Schematic of a migrating cancer cell. The processes and cell functions that can be altered by aberrant expression of formins in cancer cells are indicated. Cells, such as cancer-associated fibroblasts and immune system cells, in the surroundings of the cancerous cell influence cancer progression.

Formins have been associated with cancer by inducing epithelial-to-mesenchymal transition (EMT), migration, and invasion by their role in the actin and microtubule cytoskeletons (Figure 4, Table 1).

EMT is a cellular program by which epithelial cells progressively lose their typical polygonal appearance with segregated apical, lateral, and basal surfaces found in tightly packed cell monolayers, to adopt an elongated, mesenchymal morphology. EMT, which is normally reversible, has important roles at specific stages of embryogenesis and development [316]. At the molecular level, EMT-induced transcription factors orchestrate a profound change in gene expression. They repress the transcription of genes that maintain the epithelial state, such as those encoding E-cadherin and occludin, with the consequent disruption of cell–cell junctions. They also induce the expression of other genes that promote the mesenchymal state, such as those of N-cadherin and matrix metalloproteinases (MMPs). The malignant progression of carcinoma depends on the activation of EMT, which confers the capacity to migrate and invade neighboring tissues to neoplastic cells. Altered expression of formins affects cell morphology and can promote EMT. Examples are DIAPH3 in hepatocellular carcinoma cells [288]; FMNL1 in non-small cell lung cancer cells [293], clear cell renal cell carcinoma cells [281] and nasopharyngeal carcinoma cells [277]; FMNL2 in gastric cancer cells [312] and colorectal carcinoma cells [262]; FMNL3 in nasopharyngeal carcinoma cells and tumor xenografts [279]; and FHOD1 in lung carcinoma [294].

Migration and invasion are two cancerous processes that are closely connected with metastasis. Tumor cells can adopt two distinct modes of movement during migration in three-dimensional extracellular matrices [317,318]. One is the amoeboid mode, which allows round cells to squeeze forward through small gaps present in the extracellular matrix, which is driven by high Rho GTPase activity and actomyosin contractility in the absence of proteolytic activity. The alternative mode, called mesenchymal migration, is driven by the extension of Rac GTPase-dependent lamellipodia and requires proteolytic processing of the extracellular matrix to allow for the movement of elongated cells across wider gaps. The migration of cancer cells is generally studied in vitro by wound-healing experiments or assays in microperforated cell culture inserts in the absence of the extracellular matrix, whereas, for invasion, they are carried out in its presence (Table 1). Given that formins catalyze the formation of polymers of actin, it is not surprising that they play an important role in cancer cell migration. Examples of this function are DIAPH1-3 and DAAM1 in breast cancer [243,247], DIAPH1 in glioma [230], FMNL1 in nasopharyngeal carcinoma [277], FMNL2 in colorectal cancer [263], and FHOD1 in oral squamous carcinoma [280].

Invasion by the mesenchymal mode is facilitated by the formation of invadopodia, which are actin-based protrusions of the plasma membrane that contain metalloproteases, such as MMP2 and MMP9, that degrade the extracellular matrix [319]. DIAPH1-3 regulate invasion in cell cultures, and reduce metastasis and tumor volume in nude mice inoculated with breast cancer [243,244,245] and glioma [230]; FMNL1 does the same in glioblastoma and clear cell renal cell carcinoma cells [236,281]; FMNL2 [263] and FMNL3 [267] in colorectal cancer cells; FMNL3 in nasopharyngeal carcinoma cells [279]; and FHOD1 in oral squamous carcinoma cells [280]. As with the case of DIAPH1-3 [230,243,244], other formins could also control invasion by regulating the efficient formation of invadopodia, and the expression levels, localization and activity of metalloproteases [243].

During metastasis, tumor cells may switch between these two modes of invasion, depending on the activation status of specific Rho GTPases [317]. An interesting case is that of ovarian cancer and its relationship with DIAPH3. Exfoliated cells derived from the ovarian surface epithelium are detected as single cells, small aggregates, or highly ordered multicellular 3D spheroids. Spheroids promote ovarian cancer metastasis within the peritoneal cavity [320]. DIAPH3 expression is downregulated in early- to late-stage ovarian cancer, with this change being accompanied by disruption of the actin cytoskeleton and greater cell deformability [321,322]. While spheroids make use of both mesenchymal and amoeboid motility to invade, DIAPH3 KD disrupts spheroid architecture and enhances cell invasion by favoring ameboid migration [296]. Modulation of DIAPH3 expression also regulates the switch between the mesenchymal and ameboid migration modes in human MDA-MB-231 breast and DU145 prostate cancer cells [232,302,323]. DIAPH1 and FMNL2 have also been involved in regulating the migratory plasticity of breast tumor cell lines [241,242].

Other mechanisms by which formins can contribute to cancer progression include alteration of mitochondrial dynamics and of the microtubule cytoskeleton. An example of the former is found in INF2, which can regulate cancer progression through its role in mitochondrial fission [324,325]. Speckle-type POZ protein (SPOP), which is one of the most frequently mutated genes in prostate cancer [326], is an adapter that brings INF2 [305] and other substrates [327] to the proximity of specific E3 ubiquitin ligase complexes for ubiquitination. INF2 ubiquitination does not lead to degradation, as occurs with other proteins, but instead reduces its localization at the ER, abrogating its ability to facilitate mitochondrial fission [305], which is one of the best-characterized functions of INF2 [324,325]. A dominant negative effect probably causes SPOP mutant forms associated with prostate cancer to be defective in promoting INF2 ubiquitination, resulting in increased localization of INF2 at the endoplasmic reticulum. In turn, this facilitates augmented mitochondrial fission, which is known to promote cell migration [328], resulting in enhanced migration and invasion [305]. However, INF2 activation appears to promote apoptosis of human papillary thyroid cancer MDA-T32 cells [329]. Examples of formin levels altering cancer cells is the regulation of microtubule dynamics are the cases of DIAPH1 and DIAPH2, whose silencing impairs chromosome alignment during mitosis in colorectal cancer cell lines, giving rise to aneuploidy, and of DIAPH3, which regulates the activation of the spindle assembly checkpoint and microtubule attachment to kinetochores [174,256,258,330]. DIAPH3 downregulation increases the sensitivity of breast and prostate carcinoma cells to taxanes [138,331], which are inhibitors of microtubule dynamics.

The tumor microenvironment is an important element in cancer development. It includes cancer stem cells and the network formed by the cells, signals and extracellular matrix around the tumor [332]. Formins can promote cancer development through this network. One in vitro example is DIAPH3 in breast cancer cells, in which cancer-associated fibroblasts (CAFs) regulate tumor cell migration and invasion by secreting protein factors that reduce DIAPH3 protein levels in the tumor cells [333]. In turn, cancer-associated fibroblasts upregulate their own DIAPH3 levels to remodel the extracellular matrix and promote invasion [334]. Cancer cell inoculation models using nude mice, which are immunocompromised and often metastasize early on, do not reproduce the tumor microenvironment, which is necessary to study cancer progression under normal conditions. In contrast, genetically modified mouse models (GEMMs) develop de novo tumors in a natural immune-proficient microenvironment and are able to spontaneously progress toward metastatic disease, mimicking the process in humans. Therefore, GEMMs are of great help for basic cancer research in general, and specifically for investigating the influence of the tumor microenvironment [335,336]. Using different types of GEMMs, it was observed that enhanced levels of activin A, which is a member of the transforming growth factor β family, in the skin promote skin tumor formation and their malignant progression through the induction of a pro-tumorigenic microenvironment [337] through transcriptional activation of mDia2 in CAFs [338]. In turn, mDia2 interacts with the transcription factor p53, which is a tumor-suppressor protein [339], and prevents the accumulation of p53 in the nucleus and, consequently, p53-dependent transcriptional control of CAF-derived secreted factors that promote tumor formation [338]. These works are an example of how the use of GEMMs can help to study the role of the tumor microenvironment and the contribution of formins to this microenvironment in cancer.

Formins can also affect the tumor microenvironment through the mechanism of extracellular vesicle secretion. Thus, DIAPH3 silencing in prostate cancer cells promotes the formation of large membrane blebs that can be shed as extracellular vesicles, and the release of exosome-sized particles that enhance the proliferation of recipient tumor cells and inhibit proliferation of immune cells, thereby helping to remodel the tumor cell microenvironment [302,340]. Another mechanism is exemplified by FMNLs, whose expression is positively correlated with the levels of tumor-infiltrated immune cells in gastric cancer patients [311].

### 4.3. Formins as Prognostic Biomarkers and Therapeutical Targets in Cancer

The expression levels of some formins are associated with a favorable or unfavorable prognosis, depending on the type of cancer (Supplementary Table S10). For instance, in renal cancer, a high level of transcripts of *DIAPH1*, *DAAM1*, *FHOD3* and *FHDC1* has a favorable prognosis, whereas in *DIAPH2*, *DAAM2*, *FMNL1*, *FMNL3,* and *FHOD1*, this corresponds to an unfavorable prognosis. Some studies investigated the mRNA or protein levels of a given formin in cancer cell lines and the metastatic capacity of those cells in nude mice (Table 1). These and other studies also examined the relative levels of formin, determined by immunohistochemical analysis of biobank tumor samples relative to paired normal tissue, or levels of formin mRNA, to identify associations with the clinicopathological characteristics of the corresponding patient, such as tumor size, presence of distant metastasis, and outcome. Some examples of strong correlations are *DAAM1* in breast cancer [248]; *FMNL1* in nasopharyngeal cancer [277], non-small cell lung cancer [293], and clear cell renal cell cancer [281]; and of *FMNL3* in colorectal cancer [268].

Not only have formin levels been associated with cancer prognosis, but gene variants and deletions have also been associated with the risk of cancer progression. For example, specific *FMN1* variants have been linked to a higher risk of developing pancreatic [300] and prostate cancer [304]. In lung cancer, a variant in the FH2 domain of DAAM2 (p.Asp762Gly) has been associated with a protective effect, and, although not a statistically significant result, a variant in the DID (p.Arg172His) was found to be enriched in healthy controls [292]. The frequency of *FMN2* mutations markedly differs between the stages of colorectal cancer. This finding qualifies *FMN2* for inclusion in the list of useful biomarkers for diagnosis, pathological classification, staging and prognosis of this type of cancer [270]. *DIAPH3* deletions are common to several carcinomas and accumulate during cancer progression, as occurs in prostate, hepatocellular, and breast carcinoma. The deletions promote motility and invasion, and are associated with aggressive tumors and with metastatic prostate cancer [232].

Given their involvement in cancer, formins might have a use as therapeutic targets. Combined treatment of the formin inhibitor SIMFH2 [341] with the anti-cancer drugs taxol and cisplatin, which, respectively, stabilize microtubules and damage DNA, is useful for reducing human ovarian cancer spheroid viability in vitro [342]. However, since SIMFH2 has some off-target effects, such as reduction in the expression and activity of p53 [343] and inhibition of myosin ATPase activity [344], these should also be considered for the doses and the duration of the treatment in order to avoid harmful effects. Phenanthriplatin is a platinum (II) complex that binds to only one DNA strand (unlike cisplatin, which binds to both strands) and causes cancer-cell death by blocking gene transcription. Treatment of non-small cell lung cancer cells with phenanthriplatin, but not with cisplatin, downregulates *DIAPH2* expression, with this effect being potentially useful for treating this type of cancer [345]. Silencing DIAPH3 increases microtubule dynamics and enhances the responsiveness to taxol in prostate DU145 and LNCaP cancer cells, and low levels of *DIAPH3* mRNA improve the sensitivity to taxol and other taxanes in prostate and breast cancer cell lines. Consistent with these results, a low level of *DIAPH3* expression was correlated with improved clinical outcomes after taxane-containing chemotherapy in a clinical trial of breast cancer patients [331]. Methotrexate is a chemotherapy agent used to treat acute lymphoblastic leukemia. The ability of cells to polyglutamylate methotrexate is a major factor determining whether the drug is retained for a longer period and, consequently, its in vivo efficacy and toxicity [346]. Variants of *FHOD3* and six other genes influence the formation of methotrexate polyglutamates in leukemia treatment, making them important for methotrexate responsiveness [286].

The overexpression of *FMNL1* in blood-derived tumor cells has led to the development of novel approaches to cancer treatment, such as the use of cytotoxic T cell clones with T cell receptors specific to an FMNL1 peptide to treat lymphomas and other malignancies of cells of the immune system [347]. Mitochondrial fission is a novel target for modulating cancer development and progression because increased fission produces apoptosis [328]. Thus, treatment with interleukin-2 and the anti-tumor drug tanshinone IIA of SW480 colorectal cancer cells upregulates INF2, which increases mitochondrial fission and apoptosis [348]. This work provides a further example of how the manipulation of formin expression can be exploited to treat cancer. A better understanding of the role of the various formins in cancer could clear the way for the development of better and more specific cancer treatments in the near future.

## 5. Future Directions

Formins control not only actin filament remodeling and microtubule dynamics, but also the crosstalk between these two types of cytoskeletal structures [4,5,132,133,349]. The correct functioning of formins is essential to many cellular processes, including the formation of actin-based structures such as lamellipodia, filopodia and microvilli, and of specialized microtubule arrays, membrane trafficking, mitochondrial fission, and cell division, migration, and invasion [2,3,138,325,349]. In addition, formins can regulate gene expression, for instance, as in the case of DAAMs, by forming part of important signaling pathways [189], or, in general, by modulating the nuclear levels of the myocardin-related transcription factor coactivator (MRTF), which associates with the serum response factor (SRF), and forms an MRTF–SRF complex that controls the transcription of a myriad of genes, many of which encode proteins related to the cytoskeleton [349,350,351]. The list of MRTF-SRF target genes overlaps with gene signatures associated with cancer cell invasiveness and metastasis, response to extracellular matrix, stiffness, or response to FAK or TGFβ signaling [352,353]. Therefore, altering the activity of formins, by modulating their expression levels, or as a result of mutations in regulatory or catalytic domains, may have important consequences for the function of differentiated cells and for embryonic development and cancer. The growing list of human disorders caused by, or associated with, formin mutation illustrates the importance of formins in ensuring the normal function of cells.

An extensive analysis of multiple independent pedigrees with affected individuals is available for some cases of monogenic disorders caused by formin mutations. However, in other cases, the information is scarce and further work is needed to find more examples and mutations. The increasing use of GWS and WES approaches in Human Medical Genetics Units will certainly help identify novel variants responsible for, or associated with, pathological conditions, and novel disorders involving formin alterations. This work will facilitate diagnosis and, it is to be hoped, contribute to the development of pharmacological agents to treat these diseases.

Despite the advances in our knowledge of formins, further work is needed to fully understand the molecular mechanism leading to disease. The use of in vitro systems and animal models has undoubtedly helped us gain insights into the function of formins in health and disease. However, in most cases, the molecular mechanism by which a formin alteration leads to the pathological condition is not clear. In some cases, the primary consequence of the alteration is an increase in the actin polymerization of the affected formin, for instance, in the case of the DIAPH1 mutants with truncated carboxyl-terminal regulatory sequences. However, in cases in which the mutation maps to other formin domains, for example, the FH2 domain, the effect on the activity is less evident and requires in vitro analysis. This is also true for the pathogenic mutations in the DID of INF2, which are known to increase the actin polymerization activity of the formin. Nevertheless, despite the considerable advances, the exact mechanism underlying its activation is yet to be described. In some cases, the alteration in a formin produces disease in humans but not in mice and vice versa. The use of GEMMs will provide important insights into how formins function in vivo and how formin alterations lead to or contribute to disease, especially cancer, and hopefully to validate formin-specific drugs and develop preclinical trials to evaluate their therapeutic response.

Changes in formin levels and the emergence of formin mutations play an important role in cancer progression, although, despite the established role of formins in building invadopodia and regulating cell migration and invasion, the effect of formin alterations in cancer has yet to be fully established. Given their importance, the inclusion of formins in cancer biomarker panels might be useful for prognostic purposes. The development of new formin inhibitors with higher specificity and tolerance than SIMFH2 could be exploited to prevent chromosome segregation errors causing aneuploidy as a result of deficient mitotic spindle assembly [354]. The activation of the MRTF-SRF transcriptional complex by formins, in addition to control the expression of a large number of protein coding mRNAs, regulates the expression of non-coding RNAs. Examples of such regulation are the expression of miR-21, which promotes fibrosis and EMT transition and has been implicated in cancer, and the lncRNA Malat1/Neat2, which has been implicated in lung cancer invasion and metastasis [354]. The involvement of non-coding RNA, as well the role of extracellular vesicles preparing the niche for metastasis, are new and developing fields in cancer research [355,356]. We hope that all these combined efforts will help us fully understand the role of formins in vivo and the mechanism by which their alteration causes, or contributes to, cell dysfunction, and that they will stimulate the development of novel strategies for treating formin-related diseases.

## Figures and Tables

**Table 1:** Summary of formin expression in different types of cancer. The in vitro and in vivo experiments providing evidence of the involvement of formins in cancers and the use of formin expression as prognostic biomarkers are also summarized.

Cancer	Formin	Expression Levels (Technique) or Mutations	In Vitro				In Vivo (Nude Mice)		Prognosis	References
			Matrix Degradation	Invadopodia Formation	Migration	Invasion	Metastasis	Tumor size/Weight		
Brain	DIAPH1	↑ (IHC/T)	↑	↑	↑	↑		↑		[228,229,230]
	DIAPH1				↑					[231]
	DIAPH3									[232]
	DAAM1					↑				[233]
	DAAM1	↑ (Mass spectrometry)								[234]
	DAAM2	↑ (ISH/T)						↑		[235]
	FMNL1	↑ (IHC/T)	↑		↑	↑			Poor	[236]
	FMNL2 FMN2	Mutants								[237]
	FHOD3	↑ (WB)			↑					[238]
	FHOD3	↑ (IHC/T)			↑	↑			Poor	[239]
	FHOD1 INF2	↑ (IHC/T)			↑				Not clear	[240]
Breast	All					↑ or ↓ depending on the specific formin				[241,242]
	DIAPH1/2/3		↑	↑		↑				[243]
	DIAPH1	↑ (T/WB)	↑			↑				[244]
	DIAPH3	Deletions			↑	↑				[232]
	DIAPH3	↓ (IHC/T/WB)	↑		↑	↑				[245]
	DIAPH3									[246]
	DAAM1	↑ (IHC)		↑	↑		↑	No effect	Poor	[247,248,249,250]
	FHOD1 INF2	↑ (IHC/T)			↑	↑			No effect	[251]
Bone	DAAM1				↑					[252]
	FMNL1	↑ (T)					↑ Lung metastasis			[253]
Cervix	DAAM2	↑ (T)							Poor	[254]
Colorectal	DIAPH1	↑ (IHC)			↑	↑	↑	↑		[255]
	DIAPH1									[256]
	DIAPH2	Mutants							Poor	[257]
	DIAPH2									[258]
	DIAPH3	↑ (T) Mutants							Poor	[259]
	DAAM1/2	↑ (T)								[260]
	DAAM2	↑ (IHC/T/WB)	↑			↑			Poor	[261]
	FMNL2	↑ (IHC/T/WB)	↑	↑	↑	↑	↑	↑		[262,263,264,265,266]
	FMNL3	↑ (IHC/T/WB)	↑		↑	↑	↑	↑	Poor	[267,268]
	FMN2	↓ (T)							No effect	[269]
	FMN2	Mutants								[270]
Esophagus	DIAPH2	↓ (T)								[271]
	DAAM1					↑				[272]
Gallbladder	FMNL2	↑ (IHC)							Poor	[273]
Head & neck	DIAPH1*/2/3	↑* (IHC/T/WB) Mutants							Poor	[274]
	DIAPH2*/3	↓* (T) Mutants in DIAPH2 SNP in DIAPH3								[275]
	DIAPH2	SNP							Protective	[276]
	FMNL1	↑ (IHC/ISH/WB) Amplifications		↑	↑	↑	↑		Poor	[277]
	FMNL3	↑ (IHC/T/WB)			↑	↑			Poor	[278]
	FMNL3	↑ (IHC)	↑		↑		No effect	No effect		[279]
	FHOD1	↑ (IHC/T)	↑	↑	↑	↑				[280]
Kidney	FMNL1	↑ (IHC/T/WB)			↑	↑	↑		Poor	[281]
	DAAM2	SNP								[282]
Leukemia	DIAPH1				↑		↑			[283]
	FMNL1	↑ (T/WB)			↑			↑		[284,285]
	FHOD3	Mutants								[286]
Lymphoma	FMNL1	↑ (IHC/WB)								[287]
Liver	DIAPH3	↑ (IHC/T/WB)			↑		↑			[288]
	DIAPH3	Deletions								[232]
	DAAM2	↑ (IHC/T/WB)				↑			Poor	[289]
	FMNL2	↓ (IHC/T/WB)			↑	↑			Poor	[290]
Lung	DIAPH3	↑ (IHC/T/WB)						↑	Poor	[291]
	DAAM2	SNP							Protective	[292]
	FMNL1	↑ (IHC/T/WB)			↑	↑	↑	↑	Poor	[293]
	FHOD1				↑	↑			Poor	[294]
Ovary	DIAPH1	↑ (IHC/T/WB)							Protective	[295]
	DIAPH3				↑	↓				[296]
	FMNL1/2/3 FHOD3				↑	↑ (in vivo zebrafish)				[297]
Pancreas	DIAPH3	↑ (IHC/T/WB)				↑		↑	Poor	[298]
	FMNL1/3 FMN2	Mutants								[299]
	FMN1	SNP							Higher risk	[300]
	FMN2	↑ (T)							Protective	[301]
Prostate	DIAPH3	Deletions (IHC/ISH)			↑	↑	↑	↑		[232,302]
	DIAPH3	Knockdown			↑	↑				[246]
	FMNL3					↑				[303]
	FMN1	SNP							Higher risk	[304]
	INF2				↑	↑				[305]
Skin	DIAPH1					↑				[241]
	DAAM1	↑ (IHC)				↑	↑	↑		[306]
	FMNL2/3	↑ (IHC/WB)			↑				Poor (FMNL2)	[307]
	FMNL2	↑ (T/WB)			↑	↑				[308]
	FHOD1				↑					[309]
	FHOD1	↑ (IHC/WB)			↑	↑		↑		[310]
Stomach	FMNL1/2/3	↑ (T)							Poor (FMNL1/3) No effect (FMNL2)	[311]
	FMNL2	↑ (WB)			↑	↑				[312]
	FMNL1 FHOD1									[313]
	FHOD1	↑ (T)								[314]
Thyroid	FHOD3	↑ (T) Copy number variations								[315]

IHC, immunohistochemistry; ISH, in situ hybridization; T, transcript levels; WB, western blot; ↑/↓, High/Low. The asteisks (*) label a pair of a given formin and its corresponding expression levels.

## Data Availability

Not applicable.

## Supplementary Materials

The following are available online at www.mdpi.com/article/10.3390/cells10102554/s1. Table S1: Expression of formin genes in different human tissues. Table S2: RNA expression of formins in brain and blood cells. Supplementary Tables S3–S9: Pathogenic mutations of *DIAPH1/DIAPH2/DIAPH3/DAAM2/FMN2/INF2/FHOD3*. Table S10: Prognostic value of formin mRNA levels in cancer. Table S11: Analysis of mRNA expression of the formin genes in cancerous versus normal tissue.
